# β-Arrestin–Mediated Angiotensin II Type 1 Receptor Activation Promotes Pulmonary Vascular Remodeling in Pulmonary Hypertension

**DOI:** 10.1016/j.jacbts.2021.09.006

**Published:** 2021-11-22

**Authors:** Zhiyuan Ma, Gayathri Viswanathan, Mason Sellig, Chanpreet Jassal, Issac Choi, Aditi Garikipati, Xinyu Xiong, Nour Nazo, Sudarshan Rajagopal

**Affiliations:** a Division of Cardiology, Department of Medicine, Duke University School of Medicine, Durham, North Carolina, USA; b Trinity College of Arts and Sciences, Duke University, Durham, North Carolina, USA; c The University of North Carolina, Chapel Hill, North Carolina, USA; d Department of Biochemistry, Duke University Medical Center, Durham, North Carolina, USA

**Keywords:** angiotensin, Beta-arrestin, biased agonism, G protein–coupled receptor, pulmonary arterial hypertension, AngII, Angiotensin-2, AT1R, Angiotensin II type 1 receptor, BrdU, bromodeoxyuridine, GPCR, G protein–coupled receptor, LV, left ventricular, MCT, monocrotaline, PAH, pulmonary arterial hypertension, PASMCs, pulmonary artery smooth muscle cells, PBS, phosphate-buffered saline, PV, pressure-volume, rRNA, ribosomal RNA, RV, right ventricular, SMC, smooth muscle cell, TRV023, TRV120023

## Abstract

Pulmonary arterial hypertension (PAH) is a disease of abnormal pulmonary vascular remodeling whose medical therapies are thought to primarily act as vasodilators but also may have effects on pulmonary vascular remodeling. The angiotensin II type 1 receptor (AT_1_R) is a G protein–coupled receptor that promotes vasoconstriction through heterotrimeric G proteins but also signals via β-arrestins, which promote cardioprotective effects and vasodilation through promoting cell survival. We found that an AT1R β-arrestin-biased agonist promoted vascular remodeling and worsened PAH, suggesting that the primary benefit of current PAH therapies is through pulmonary vascular reverse remodeling in addition to their vasodilation.

## Highlights

• We tested the effects of a β-arrestin–biased agonist (TRV023) of the angiotensin II (AngII) type 1 receptor (AT_1_R), which acts as a vasodilator while not blocking cellular proliferation, compared to a balanced agonist, AngII, and an antagonist, losartan, in PAH.
• In acute infusion, AngII increased right ventricular pressures while TRV023 and losartan did not. However, in chronic infusion in monocrotaline PAH rats, both TRV023 and AngII had significantly worse survival than losartan.
• Both TRV023 and AngII enhanced proliferation and migration of pulmonary artery smooth muscle cells from patients with PAH.
• β-arrestin-mediated AT_1_R signaling promotes vascular remodeling and worsens PAH, and suggests that the benefit of current PAH therapies is primarily through pulmonary vascular reverse remodeling.

## Visual Abstract

**Figure:**
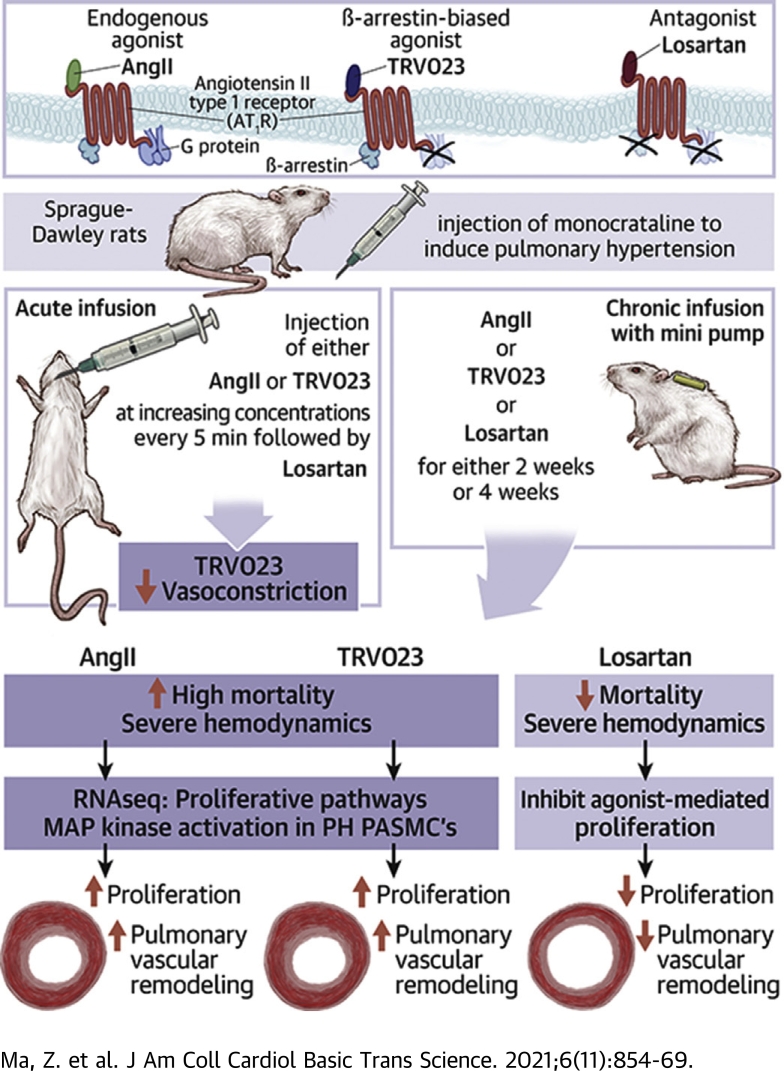

Pulmonary arterial hypertension (PAH) is a disease associated with excessive pulmonary vascular remodeling characterized by dysfunction of endothelial cells and proliferation of smooth muscle cells that leads to obliteration of pulmonary arterioles. This results in a high pulmonary vascular resistance, right ventricular (RV) hypertrophy, dilation, and ultimately failure (1). Current PAH therapies largely target vasoactive mediators such as endothelin-1 and prostacyclin, which signal through G protein–coupled receptors such as the type A endothelin receptor and prostacyclin receptor, respectively (2). However, the utility of these therapies is thought to be limited in their efficacy by acting primarily as pulmonary vasodilators and not affecting the pulmonary vascular remodeling that underlies progression of the disease (3). Other vasoactive mediators, such as angiotensin II (AngII), are also thought to contribute to the pathobiology of PAH. The expression of AngII and AT_1_R is elevated in PAH and increased levels of AngII are associated with PAH progression and mortality (4, 5, 6, 7). Blocking AT_1_R signaling is beneficial in rats with PAH, including restored RV dysfunction, decreased pulmonary vascular remodeling, and delayed PAH progression (4,8).

G protein–coupled receptors (GPCRs), such as the AT_1_R, signal canonically through heterotrimeric G proteins and multifunctional β-arrestin adapter proteins, which in addition to signaling, also promote receptor internalization and desensitization of G protein signaling (9). Subsequently, G protein–dependent (10) and/or β-arrestin–dependent (11) signaling is transduced by the activation of downstream mediators, such as Ca^2+^-dependent protein kinase C, mitogen-activated protein kinase signaling, and GPCR-mediated transactivation of receptor tyrosine kinases (12,13). G protein–mediated AT_1_R signaling has been found to lead to hypertension, myocardial hypertrophy, and cardiac dysfunction (13,14), whereas β-arrestin–mediated signaling has been shown to promote cardioprotective effects and decrease systemic and renovascular resistance in systolic heart failure (15, 16, 17, 18). Recently, “biased” AT_1_R ligands were shown to engage and stabilize distinct active conformations of AT_1_R (19) and activate only a subset of receptor-mediated signaling pathways (15,20), such as only through G proteins (“G protein–biased”) or β-arrestins (“β-arrestin–biased”) (21,22). For example, compounds such as TRV120027 and TRV120023 (TRV023) have been shown to function as β-arrestin–biased agonists, antagonizing G protein signaling and activating β-arrestin–mediated signaling. These drugs act acutely as vasodilators, by antagonizing AT_1_R G protein–mediated vasoconstriction, while promoting cardiac function and decreasing apoptosis through their β-arrestin–mediated signaling.

It is not known whether the benefit of current therapies is primarily mediated through vasodilation or reverse remodeling of the pulmonary vasculature, as patients on long-standing PAH therapies can still display severe vascular pathology (23). We hypothesized that we could address this question through the selective activation of AT_1_R β-arrestin–mediated signaling with a β-arrestin–biased agonist, which would be predicted to act as an acute vasodilator while promoting AT_1_R β-arrestin–mediated signaling. We compared the physiological and molecular effects of a β-arrestin–biased AT_1_R agonist, TRV023, to a balanced agonist, AngII, and an antagonist, losartan, in the treatment of PAH. We found that β-arrestin–biased AT_1_R stimulation promoted pulmonary vascular remodeling, demonstrating that vasodilation in the presence of β-arrestin-biased AT_1_R activation worsens PAH. These findings suggest that the beneficial long-term effects of drugs targeting GPCR signaling in PAH is not through vasodilation but from reversing pulmonary vascular remodeling.

## Methods

### AT_1_R ligands

AngII and TRV023 (Sar-Arg-Val-Tyr-Lys-His-Pro-Ala-OH) were synthesized by GenScript USA with quality control assessed by high-performance chromatography and mass spectrometry. Losartan was purchased from Santa Cruz Biotechnology (sc-204796). AngII was also obtained from Sigma (A9525-1MG).

### Animals

All animal experiments were conducted in compliance with institutional guidelines and were approved by the Duke University Institutional Animal Care and Use Committee (Protocol: A175-16-08). Male Sprague-Dawley rats (5-6 weeks old, Charles River Laboratories) weighing between 150 and 200 g were used in the chronic infusion studies. For acute infusion, 8- to 10-week-old Sprague-Dawley rats with an average weight of 300 g were used.

### Experimental protocols for PAH treatment

Pulmonary hypertension was induced by a single subcutaneous injection of crotaline (MCT, 60 mg·kg^−1^; Sigma). For chronic treatments, either vehicle (phosphate-buffered saline [PBS]), 1 mg·kg^−1^·d^−1^ AngII, 10 mg·kg^−1^·d^−1^ losartan, or 14.4 mg·kg^−1^·d^−1^ (10 μg·kg^−1^·min^−1^) TRV023 were delivered by Alzet osmotic minipumps (Model 2ML4 or 2ML2) placed in a subcutaneous pouch under anesthesia with isoflurane. These doses were based on a previous study in rats with AngII and TRV023 (24). Osmotic pumps were implanted 2 weeks after MCT injection for a duration of 4 weeks for survival studies and 1 week after MCT injection for a duration of 2 weeks for histology and hemodynamic analyses.

### Hemodynamic analysis

For acute hemodynamic responses, the level of anesthesia was regulated by delivery of 3% isoflurane administered through a nose cone with 100% O_2_. Rats were placed on a servo-controlled heating table to maintain rectal temperature constant at 37°C. A midline skin incision was made to expose the trachea, carotid artery, and jugular vein. A pressure transducer–tipped curved 2-French catheter (SPR-513; Millar) was inserted into the right ventricle (RV) via right internal jugular vein access, a pressure-volume (PV) catheter was inserted into the aorta and left ventricle (LV) via the left carotid artery (SPR-869; Millar), and the left internal jugular vein was accessed for the administration of intravenous fluids and drugs. After obtaining vascular access, the animal was allowed to stabilize for 15 minutes before the beginning of the experimental protocol. Rats (n = 6) were infused with increasing doses of AngII or TRV023 followed by treatment with losartan. Dosages of AngII and TRV023 were determined based on previous studies (24,25). At 5-minute intervals, either increasing doses (1, 10, and 100 μg/kg) of AngII or increasing doses (10, 100 μg/kg, 1 mg/kg) of TRV023 were administered followed by treatment with 100 μg/kg losartan via the left internal jugular vein at a volume of 1 μL/g body weight (25-30 μL total volume) followed immediately by 30 μL of normal saline. Before the injection of vasoactive agents, each rat received an equivalent volume (55-60 μL, 2 μL/g body weight) of normal saline as a vehicle control. Peak rates of LV and RV pressure rise (dP/dtmax) and fall (dP/dtmin) were determined. RV and LV pressures were measured at end-diastole (EDRVP and EDLVP, respectively) and at peak-systole (RVPmax and LVPmax). After the procedure, the rats were euthanized by bilateral thoracotomy. Data were analyzed in LabChart (ADI Instruments).

For chronic hemodynamic responses, the open-chest approach for RV heart catheterization was performed as previously described (26). Rats were anesthetized with xylazine (2.5 mg/kg) and ketamine (10 mg/kg**).** A needle was inserted into the trachea to serve as endotracheal intubation, the cannula connected to a volume cycled rodent ventilator on normal air with a tidal volume of 1.2 mL and respiratory rate of 82/min. The conductance catheter tip was inserted through a stab wound on the apical RV free wall until all electrodes were inside the ventricle. RV preloads was altered by tightening a suture placed around the interior vena cava to perform RV PV loop recordings.

RV hypertrophy was quantified as the ratio of RV to body weight (RV/BW). Similarly, LV hypertrophy was determined as the ratio of LV and septal weight to body weight ([LV+S]/BW)*.* Measurements were performed by investigators blinded to the experimental groups.

### Immunofluorescence and morphometric analysis

Immunofluorescence and morphometric quantification were performed as previously reported (27). Briefly, lung tissues were paraffin-embedded and sectioned at 5 μm. Sections were incubated at 4°C with anti–Von Willebrand factor (1:200; Dako) and anti–α-smooth muscle actin (1:100; Sigma) antibodies in 10% goat norm serum in PBS overnight, followed by incubation with Alexa Fluor 488 goat anti-mouse or Alexa Fluor 594 goat anti-rabbit secondary antibody for 1 hour in the dark. Images were acquired using an LSM upright 780 confocal microscope. For the morphometric analyses, 10 random fields were examined for 20 to 80 μm muscular arteries. The external and internal media perimeters of muscular arteries were measured using ImageJ and external and internal media radii were calculated using r = perimeter/2π. The medial wall thickness was expressed as (external media radii – internal media radii)/external media radii. For costaining, lung tissues were paraffin-embedded and sectioned at 5 μm. Sections were incubated at 4°C with anti-Ki67 Alexa Fluor 488 (1:50; Cell Signaling) and anti–α-smooth muscle actin Cy3 (1:100; Sigma) antibodies in 10% normal goat serum in PBS overnight. DNA was stained with 4′,6-diamidino-2-phenylindole (DAPI). For the morphometric analyses, 10 random fields were examined for 50 to 100 μm muscular arteries. Using LAS X software costaining of Ki-67 with α-smooth muscle actin–positive cells to total number of cell ratios was determined in the pulmonary arteries. Quantifications were performed by investigators blinded to the experimental groups.

### Immunohistochemistry

Immunohistochemistry for lung tissues from MCT rats was performed by the Department of Pathology, Duke University Medical Center. Briefly, antigen was retrieved at 100°C for 20 minutes in pH 6.1 citrate buffer. Lung sections were stained for Ki-67 (RM-9106-S, 1:400; Thermo Scientific) and Cleaved Caspase-3 (9661s, 1:400; Cell Signaling Technology). To assess proliferative and apoptosis markers in pulmonary arteries of MCT rats treated with AngII or TRV023 or losartan, 5 random fields were examined for 50- to 80-μm arteries from 5 rats for each experimental group. Using ZEN lite software, Ki-67 and cleaved caspase-3 positive cells to total number of cell ratios were determined in the pulmonary arteries. Quantifications were performed by investigators blinded to the experimental groups.

### Human samples

Human pulmonary arterial smooth muscle cells (PASMCs) were isolated by culture from explanted lung tissue from confirmed subjects with PAH undergoing lung transplantation at Duke University Medical Center. The study protocol for human tissue donation was approved by the Institutional Review Board of Duke University Medical Center (Pro00044369) and written informed consent was obtained from each subject. Control PASMCs were obtained from human lung tissues from normal donor lungs at the Cleveland Clinic (gift of Drs Suzy Comhair and Serpil Erzurum) and from pooled primary cells from a commercial sources (Lonza).

Pulmonary arteries were minced and digested overnight in 30 mL of digesting solution (Hank’s Balanced Salt Solution, collagenase (0.1 mg/mL), DNase (0.1 mg/mL), HEPES (2.5 mL), penicillin/streptomycin (250 mg/mL), amphotericin B (0.625 mg/mL) and 1% anti-anti). Cells were then filtered using 100-μm pore nylon cell strainer and neutralized with 10 mL smooth muscle cell (SMC) growth media. Cells were centrifuged at 330*g* for 7 minutes and resuspended with SMC growth medium. Cells were cultured in SMC medium (310-500; Cell Applications) containing growth supplements (311-GS; Cell Applications), 1% anti-anti, and 1% penicillin/streptomycin.

### Bromodeoxyuridine proliferation assay

Cell proliferation was assessed by the Cell Proliferation Enzyme-Linked Immunosorbent Assay Bromodeoxyuridine (BrdU) kit (11647229001; Roche) according to the manufacturer’s instructions. Approximately 8,000 PASMCs were seeded in each well of a 96-well plate. At 60% to 70% confluence, cells were starved overnight and stimulation with 100 nM AngII for 24 hours and 1 μM TRV023 for 48 hours. BrdU was added to the cells during the last 6 to 8 hours of incubation. Cells were then fixed for 30 minutes and probed with anti-BrdU-POD antibody for 90 minutes followed by substrate development. The chemiluminescence was quantified by measuring the absorbance at 370 nm and 492 nm using a BioTek Synergy Neo 2 multi-mode reader.

### In vitro scratch assay

PASMCs were seeded on 24-well plates. At 80% to 90% confluency, cells were serum starved for 24 hours and scratches were made using a 20-μL pipette tip. Cells were washed with Dulbecco’s PBS (without calcium and magnesium) and stimulated with basal smooth muscle cell medium containing 1 μM AngII, 10 μM TRV023 with or without 10 μM losartan. The wound closure was monitored using a live-cell station Zeiss Axio Observer microscope (Duke Light Microscopy Core Facility). The images were captured hourly in real time from 0 to 12 hours. The initial edges of the scratch at 0 hour were marked and migrated distance at 12 hours was measured using MetaMorph Premier (Molecular Devices).

### Protein isolation and immunoblotting

Human PASMCs were serum starved for 24 hours and treated with increasing concentration of AngII or TRV023. The lysates were collected 10 and 20 minutes after treatment using 1X sodium dodecyl sulfate (SDS) buffer. The proteins were denatured at 100°C for 10 minutes. The lysates were stored at −20°C. Total proteins extracted with 1X SDS were separated by 10% SDS–polyacrylamide gel electrophoresis and transferred to nitrocellulose membranes (1620112; Bio-Rad). The membranes were blocked in 5% bovine serum albumin for 1 hour at room temperature and probed with primary antibodies, extracellular signal-regulated kinase (ERK) (06-182; Millipore Sigma), phospho-ERK (8201S; Cell Signaling), p38 (8690S; Cell Signaling), and phospho-p38 (4511S; Cell Signaling) at 4°C overnight. The membranes were incubated with secondary antibodies for 1 hour at room temperature. The blots were developed using SuperSignal West Pico PLUS Chemiluminescent Substrate (34580; Thermo Fisher Scientific) and captured by Bio-Rad ChemiDoc Imaging System. The data analysis was performed using ImageLab software (Bio-Rad).

### Next-generation sequencing (RNA sequencing)

Total RNA was extracted from approximately 10 mg of MCT rat lung tissues using the RNeasy Fibrous Tissue Mini Kit (74704; Qiagen) following the manufacturer’s instructions. Total RNA was submitted to Novogene Corporation, Inc., for quality analysis, RNA sequencing, and bioinformatics analysis. All RNA samples had RNA integrity numbers approximately 8 to 9. All qualified samples were processed for library construction followed by sequencing. Reads were aligned to the rat genome and differential gene expression analysis was obtained. The Database for Annotation, Visualization and Integrated Discovery (DAVID) v6.8 was used to analyze the signaling pathways using *Rattus norvegicus* as background.

### RNA isolation

The PASMCs isolated from patients with pulmonary hypertension (PH) were treated with 500 nM AngII and 5 μM TRV023 for 4 hours. The total RNA from PASMCs were isolated by using Qiagen RNeasy Plus Mini/Micro kit. The procedures were followed according to the manufacturer’s instructions. Finally, RNA was dissolved in diethylpyrocarbonate (DEPC) water and stored at −80°C. The quality and concentration of total RNA was measured by a NanoDrop spectrophotometer. Total RNA of 1 μg was reverse transcribed into complementary DNA (cDNA) using the Bio-Rad iScript cDNA synthesis kit according to the manufacturer’s instructions.

### Quantitative polymerase chain reaction

The cDNA was mixed with iTaqTM SYBR Green Supermix and the relevant primers. The human polymerase chain reaction (PCR) primers for 18S ribosomal RNA (rRNA), matrix metalloproteinase (MMP)-1, MMP-2, MMP-7, MMP-9, tissue inhibitor of metallopeptidases (TIMP)-1, TIMP-2, TIMP-3 and TIMP-4 are shown in Supplemental Table 1. The rat PCR primers for 18S rRNA, GDF15, Cxcl14, and Arpp19 are shown in Supplemental Table 2. The cDNA levels were measured using the Applied Biosystems 7300 Real-Time PCR System. The PCR was performed by initial incubation at 50°C for 2 minutes, denaturing at 95°C for 30 seconds, then 40 cycles of denaturing at 95°C for 15 seconds and annealing at 60°C for 1 minute. Data are expressed as the relative expression (fold change = 2−ΔΔCt) of each target gene compared with 18S rRNA.

### Statistics

All graphs and data generated in this study were analyzed using GraphPad Prism 8 Software. All quantitative data are presented as mean ± standard error of the mean (SEM). The statistical significance of differences was determined using Student’s 2-tailed *t*-test in 2 groups, and 1-way or 2-way analysis of variance along with ad hoc pairwise comparisons using Bonferroni's (select contrasts), Tukey's (all comparisons), or Sidak's (comparisons to a control) multiple comparison test. Survival curves were analyzed by the Kaplan-Meier method and compared by a log-rank test. A *P* value < 0.05 was considered statistically significant.

## Results

### Acute infusion of β-arrestin–biased AT_1_R agonist does not increase LV or RV pressure

**Figure 1:**
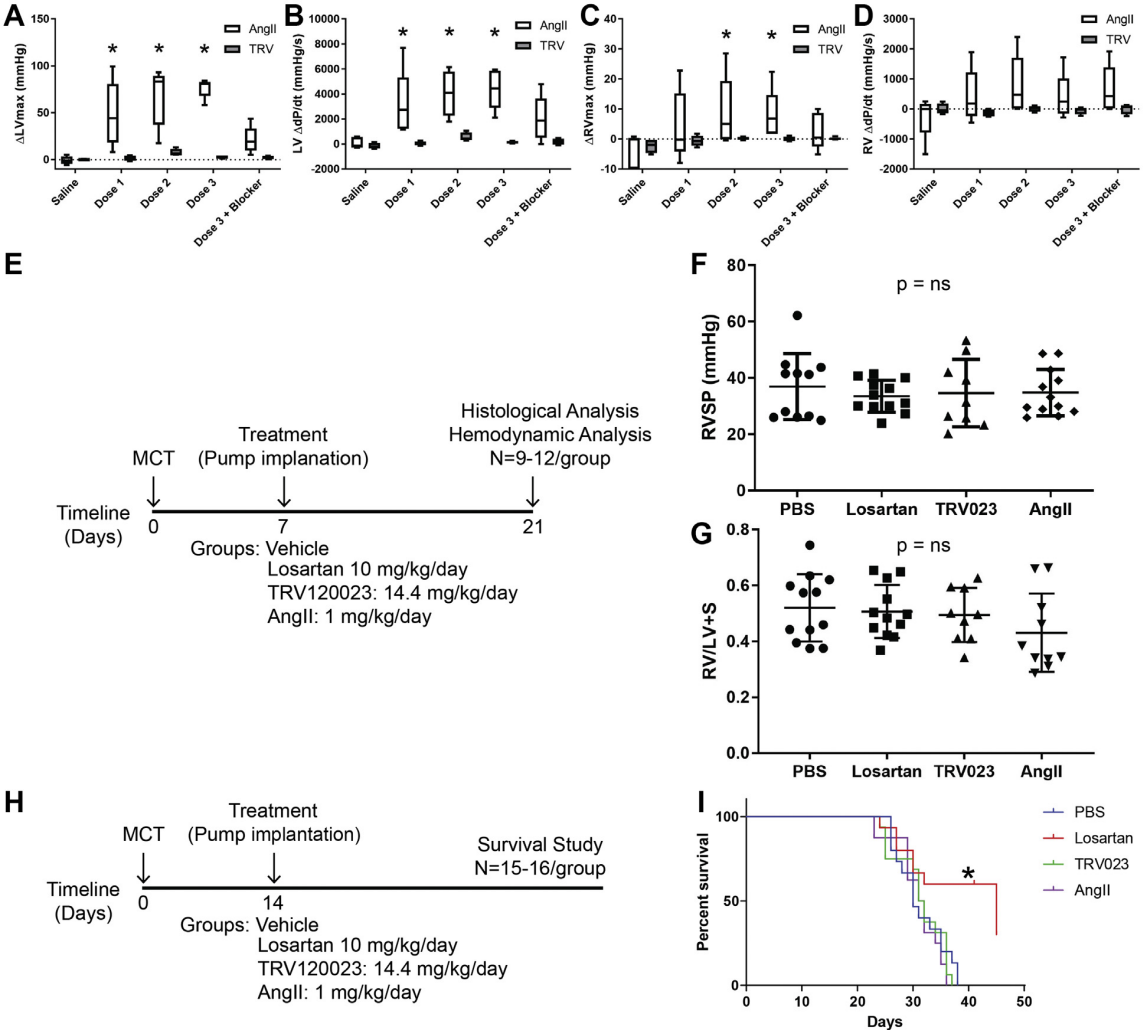
Hemodynamic Effects of Targeting AT_1_R With Acute or Chronic Infusions in Healthy and MCT PH Rats At 5-minute intervals, either increasing doses (1, 10, and 100 μg/kg) of AngII or increasing doses (10, 100 μg/kg, 1 mg/kg) of TRV023 were administered followed by treatment with 100 μg/kg losartan via the left internal jugular vein. Acute infusion of AngII in healthy Sprague-Dawley rats resulted in significant increases in left ventricular (LV) **(A)** pressure and **(B)** contractility (dP/dt – change in pressure over change in time), whereas TRV023 did not. AngII infusion also increased right ventricular (RV) **(C)** pressure but not **(D)** contractility, whereas TRV023 did not have a significant effect on either. “Blocker” denotes losartan. With chronic infusion in MCT PH rats, 1 week after MCT injection a **(E)** 2-week infusion of AngII, TRV023, losartan, or vehicle did not result in significant changes in **(F)** RV systolic pressure (RVSP) or **(G)** RV hypertrophy as assessed by Fulton index (RV mass/LV + septum (S) mass). Assessing survival by Kaplan-Meier in MCT PH rats **(H)**, 2 weeks after MCT injection **(I)** an infusion of losartan had a significant effect on survival, whereas the AngII, TRV023, and vehicle infusions had no effect. ∗*P* < 0.05. AngII = angiotensin II; AT_1_R = angiotensin II type 1 receptor; MCT = monocrotaline; PH = pulmonary hypertension; TRV023 = TRV120023.

On stimulation by the endogenous ligand AngII, AT_1_R activates both heterotrimeric G proteins and β-arrestin adapter proteins. Stimulation with the β-arrestin–biased agonist TRV023 has been reported to only robustly recruit β-arrestin in the absence of G protein activation, enhancing myocyte contractility but without promoting hypertrophy, as seen with AngII (24). Because AngII and TRV023 activate distinct signaling pathways through AT_1_R, we first set out to examine the physiological effects of AngII or TRV023 on LV and RV hemodynamics with acute infusion of drug in healthy Sprague-Dawley rats while monitoring their hemodynamics with simultaneous right and left heart catheterization. Acute infusion of AngII in normal rats increased both LV pressure and contractility (dP/dt), whereas TRV023 did not (Figures 1A and 1B). Similarly, AngII increased RV pressure, whereas TRV023 did not (Figure 1C), and neither drug had a significant effect on RV contractility (Figure 1D). Notably, neither AngII or TRV023 had a significant effect on LV filling pressure (data not shown). These findings are consistent with G protein–mediated AT_1_R signaling promoting both systemic and pulmonary vasoconstriction. Importantly, blocking both β-arrestin and G-protein–mediated AT_1_R signaling by the administration of losartan completely reversed AngII-induced elevated LV pressure.

### β-arrestin–mediated AT_1_R activation does not improve hemodynamics in PAH rats in chronic infusion

To determine how β-arrestin–biased AT_1_R signaling affects hemodynamics in PAH, we treated MCT PAH rats 1 week after MCT injection with minipumps of PBS, AngII, TRV023, or losartan for 2 weeks followed by an analysis of hemodynamics with RV PV loop analysis (Figure 1E). At this time point after MCT treatment, we would expect the rats to demonstrate pulmonary vascular pathology with compensated cardiac function without significant mortality (28). In contrast to acute infusion, chronic treatment of MCT PAH rats with TRV023 or losartan did not result in any significant improvement in RV systolic pressure compared with either PBS or AngII treatment (Figure 1F). Similarly, there were no significant differences in most hemodynamic parameters from PV loop analysis (Supplemental Figure 1). Since there was no effect of chronic infusion of AngII, TRV023, or losartan on RV hemodynamics in MCT PAH rats, we next assessed RV and LV hypertrophy. We found that LV hypertrophy was increased in rats that received TRV023 or AngII compared with losartan (Supplemental Figure 2), consistent with the significant effect of AngII on LV hemodynamics, whereas there were no differences in RV hypertrophy among treatment groups (Figure 1G). Although this was largely consistent with previous studies demonstrating no significant hemodynamic benefit of AT_1_R antagonism in MCT PAH (4), it suggested that the previously described beneficial effects of AT_1_R antagonism in PAH were not directly mediated through an improvement in RV hemodynamics.

### Blocking both β-arrestin and G protein–mediated AT1R signaling improves survival in MCT PAH

The MCT PAH model in rats is characterized by approximately 70% mortality 4 weeks after treatment with MCT (29). To determine whether β-arrestin–mediated AT_1_R activation had beneficial effects on survival, rats were injected with MCT to induce PAH, and after 2 weeks minipumps were implanted for treatment with AngII, TRV023, or losartan for up to 4 weeks via minipump (Figure 1H). Compared with the vehicle-treated group, losartan treatment markedly improved survival compared with AngII, TRV023, and PBS (Figure 1I). Notably, AngII treatment did not worsen survival relative to PBS, which may be due to high levels of AT_1_R activation in vehicle conditions from higher levels of circulating AngII, as has been observed in patients with PAH (4). These results demonstrate that antagonizing both β-arrestin and G protein–dependent AT_1_R signaling is beneficial in PAH, but the selective inhibition of G protein–dependent signaling with a β-arrestin–biased agonist (TRV023) did not improve hemodynamics or outcomes in PAH. This suggests that β-arrestin–mediated AT_1_R signaling contributes to PAH pathology.

### β-arrestin–mediated AT_1_R signaling stimulates cell proliferation in vivo

**Figure 2:**
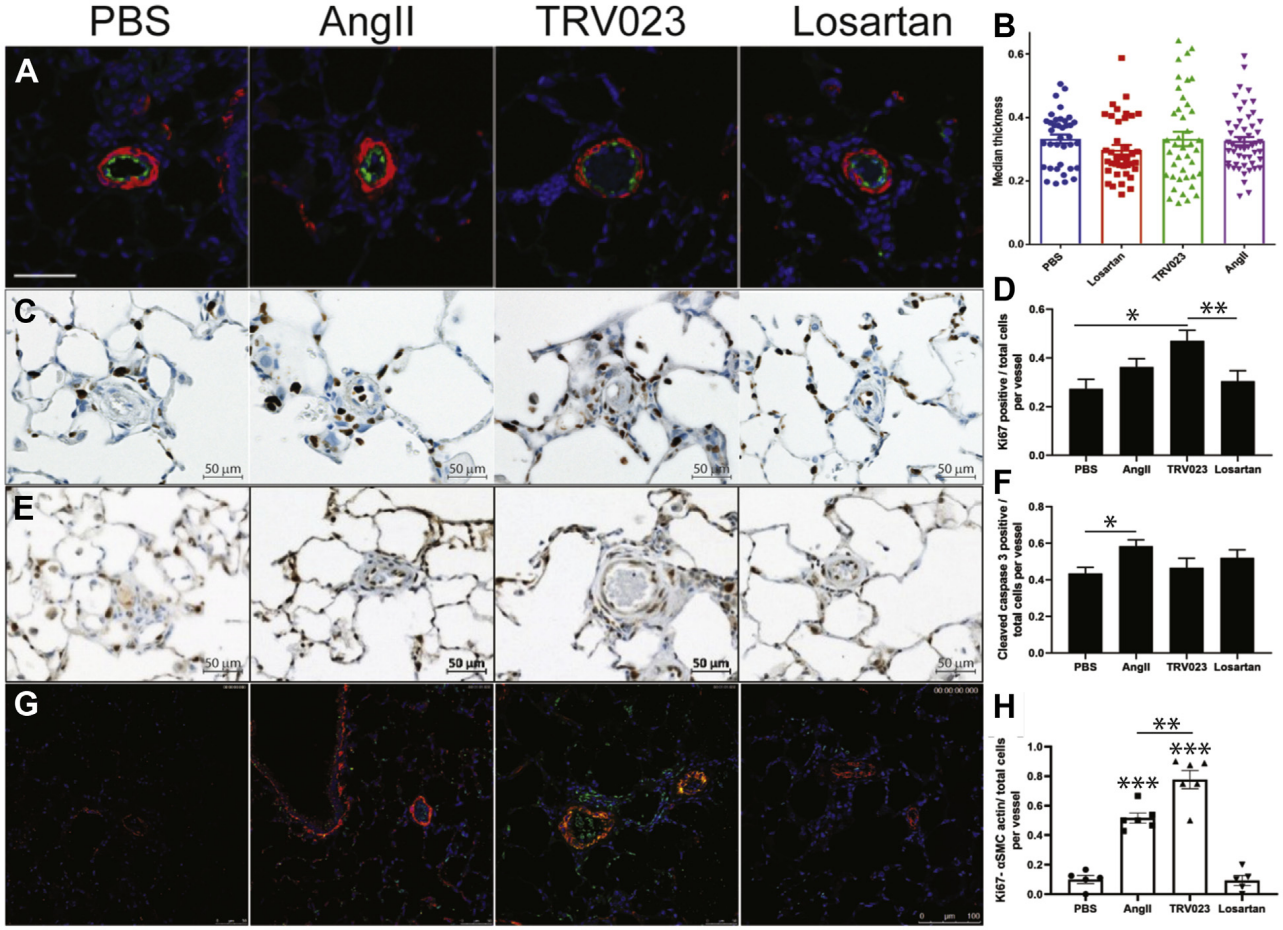
Effect of TRV023 on Cellular Proliferation in the Lungs of MCT Rats Lungs from MCT rats treated with AngII, TRV023, losartan, or vehicle were inflation-fixed in 10% formalin, paraffin-embedded, and then sectioned for staining. **(A)** Endothelial cell staining with vWF **(green)** and α-smooth muscle cell actin **(red)** demonstrated **(B)** no significant differences in gross pulmonary vascular remodeling. For immunohistochemical staining with Ki-67 and cleaved caspase-3, the number of Ki-67/cleaved caspase-3–positive **(brown)** and Ki-67/cleaved caspase-3–negative cells **(blue)** were counted in pulmonary arteries and the ratio of positive/total cells was calculated. Representative micrographs showing that **(C)** Ki-67 and **(E)** cleaved caspase-3–positive cells were stained in **brown** and the negative cells in **blue**. Quantitative assessment of **(D)** Ki-67– and **(F)** cleaved caspase-3–positive cells from MCT rats. n = 5 rats for each group. N = 5 PA for each rat. Each bar represents mean ± SEM (n = 25). ∗*P* < 0.05; ∗∗*P* < 0.01. Scale = 50 μm. **(G)** Immunofluorescence demonstrating colocalization of Ki-67 **(green)** and α-smooth muscle actin **(red)** along with 4′,6-diamidino-2-phenylindole (DAPI) **(blue)** in AngII- and TRV023-treated samples. **(H)** Assessment of Ki-67 signal with α-smooth muscle cell actin. ∗∗∗*P* < 0.001 from PBS- and losartan-treated samples. ∗∗*P* < 0.01 between TRV023- and AngII-treated samples. αSMC = α-smooth muscle cell; PBS = phosphate-buffered saline; vWF = von Willebrand factor; other abbreviations as in Figure 1.

As we did not observe significant hemodynamic changes between groups, although there was a significant difference in survival, we next examined the pathology of lungs in MCT PAH rats treated with AngII, TRV023, losartan, and PBS. Consistent with no changes in the measured RV hemodynamics, there were no significant differences in the medial wall thickness of small pulmonary arteries in rats treated with PBS, AngII, TRV023, or losartan (Figures 2A and 2B); however, Ki-67–positive cells in vascular walls were significantly increased in rats that received TRV023 compared with PBS or losartan (Figures 2C and 2D). Moreover, AngII infusion significantly increased proapoptotic activity as assessed by cleaved caspase-3 staining compared with control, but not compared with TRV023, which may partially explain why AngII infusion did not increase the number of Ki-67–positive cells (Figures 2E and 2F). The increased Ki-67 signal in smooth muscle cells was also demonstrated by immunofluorescence, demonstrating colocalization of Ki-67 with α-smooth muscle cell actin in the AngII- and TRV023-treated conditions, although Ki-67 levels were more significantly increased in the TRV023- compared with AngII-treated samples (Figures 2G and 2H). Colocalization of Ki-67 with SMA was confirmed in single channel images for each replicate (Supplemental Figure 3). Taken together, these findings suggest that β-arrestin–mediated AT_1_R signaling by TRV023 promotes specific aspects of adverse pulmonary vascular remodeling through stimulating cell proliferation, whereas the effects of AngII predominantly affect apoptosis.

### ANGII and TRV023 activate transcriptional programs for pulmonary vascular remodeling

**Figure 3:**
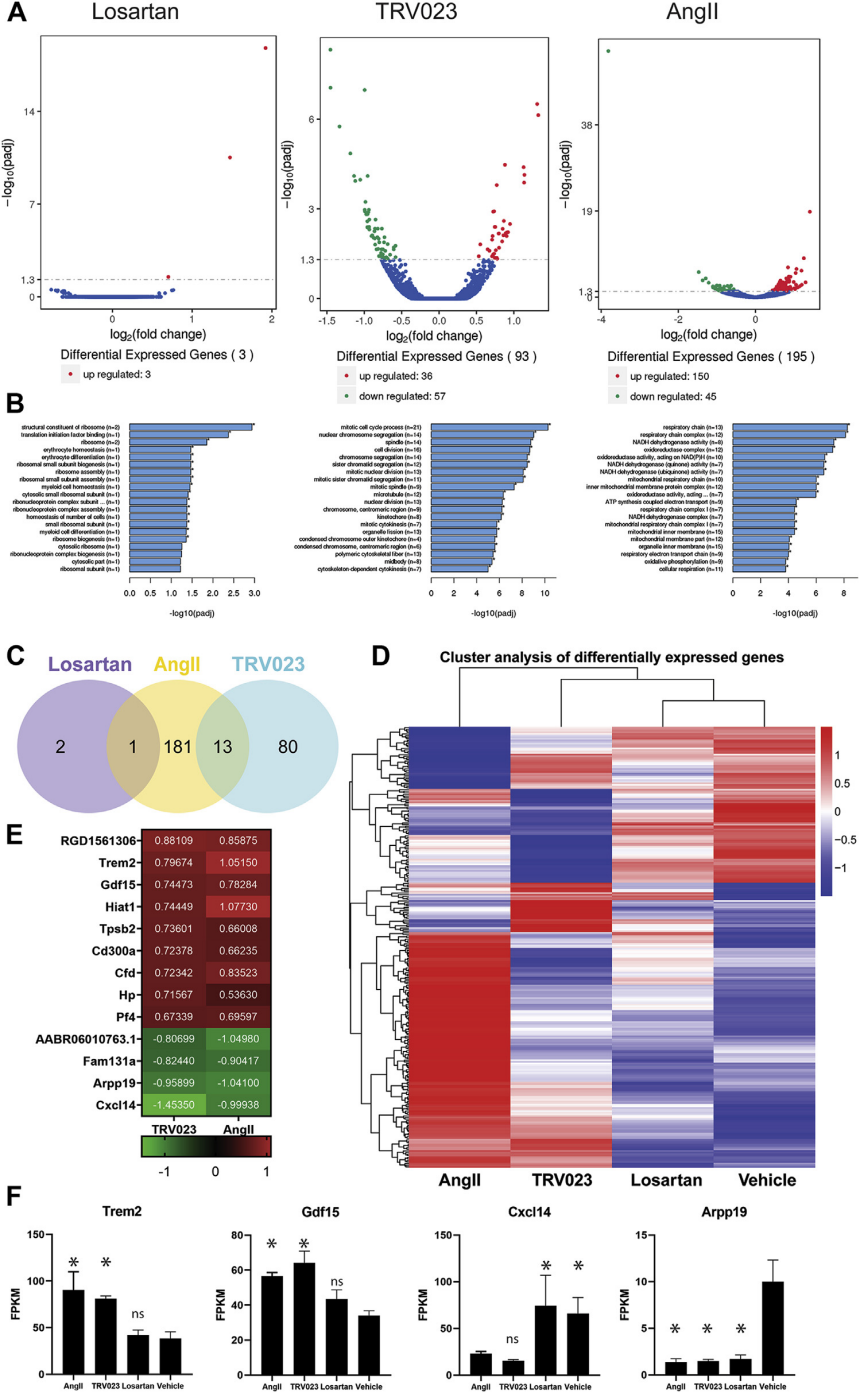
RNA Sequencing of Lungs From AT_1_R Ligand–Treated MCT Rats Demonstrates That TRV023 and AngII Regulate Pathways Important in **Proliferation and Metabolism** Total RNA from lung tissues from MCT rats treated with or without AngII, TRV023, losartan, and vehicle were isolated, and RNA sequencing was performed (n = 3 rats per group). **(A)** Volcano plot of differentially expressed genes between losartan, TRV023, and AngII from vehicle. **(B)** Gene ontology (GO) pathways from those differentially expressed genes for losartan, TRV023, and AngII, respectively. **(C)** Venn diagram of genes that were differentially expressed relative to vehicle in the losartan, TRV023, and AngII groups. **(D)** Hierarchical clustering of differentially expressed genes between AngII, TRV023, losartan, and vehicle. **(E)** Heat map showing selected differentially expressed genes shared between AngII- or TRV023-treated MCT rats. **(F)** Increased messenger RNA (mRNA) expression of MAPK signal regulating candidates *Trem2* and *Gdf15* by RNA sequencing. Decreased mRNA expression of negatively MAPK regulating candidates *Cxcl14* and *Arpp19* by RNA sequencing. Pathway enrichment performed by DAVID and GO enrichment tool. Statistical analysis was performed by one-way ANOVA. ∗*P* < 0.05 versus vehicle-treated MCT rats. ANOVA = analysis of variance; DAVID = Database for Annotation, Visualization and Integrated Discovery; FPKM = fragments per kilobase of exon model per million reads mapped; MAPK = mitogen-activated protein kinase; other abbreviations as in Figure 1.

To identify the signaling pathways that may underlie disease progression in AT_1_R ligand–treated rats, we performed lung transcriptome analysis from MCT rats treated with AngII, TRV023, losartan, and PBS with RNA sequencing. Comparing drug-treated with vehicle-treated groups, only 3 genes were differentially regulated between vehicle and losartan, compared with 93 genes differentially regulated between vehicle and TRV023, and 195 genes differentially regulated between vehicle and AngII (Figure 3A). We performed gene ontology enrichment to identify specific cellular processes associated with these differentially regulated genes, which were consistent with TRV023 and AngII promoting changes in cell proliferation and cell energetics, respectively (Figure 3B). Only 13 differentially expressed transcripts were shared between the AngII and TRV023 groups (Figure 3C), although hierarchical clustering of the differentially expressed genes demonstrated that the AngII and TRV023 groups were more similar to each other than to the losartan and vehicle groups (Figure 3D). To identify the underlying pathways common to the 13 genes that were regulated in common in the AngII and TRV023 groups, we used the DAVID bioinformatics database and PANTHER analysis. Among those 13 genes that were regulated in common between AngII and TRV023 (Figure 3E), these cell signaling pathway enrichment analysis tools revealed upregulation of transcripts in AngII and TRV023 MCT lungs that are known to be regulated by mitogen-activated protein kinase (MAPK) signaling cascade, including triggering receptor expressed on myeloid cells 2 (Trem2) (30) and growth differentiation factor 15 (Gdf15) (31,32) (Figure 3F). The RNA-sequencing findings were confirmed with quantitative PCR for 3 of these markers (Supplemental Figure 4). Activation/phosphorylation of MAPK isotypes, such as ERKs, MAPK 14 (p-38), and MAPK 8 (JNK), are known to regulate in vascular smooth muscle cell proliferation, extracellular matrix production, and migration (33, 34, 35). In our transcriptome analysis we also found a phosphatase inhibitor, cAMP-regulated phosphoprotein 19 (Arpp19) that is downregulated in both Angiotensin-2 and TRV023 MCT rat lungs (Figure 3F).

### β-arrestin–mediated AT_1_R signaling promotes pathways important in vascular proliferation and remodeling

**Figure 4:**
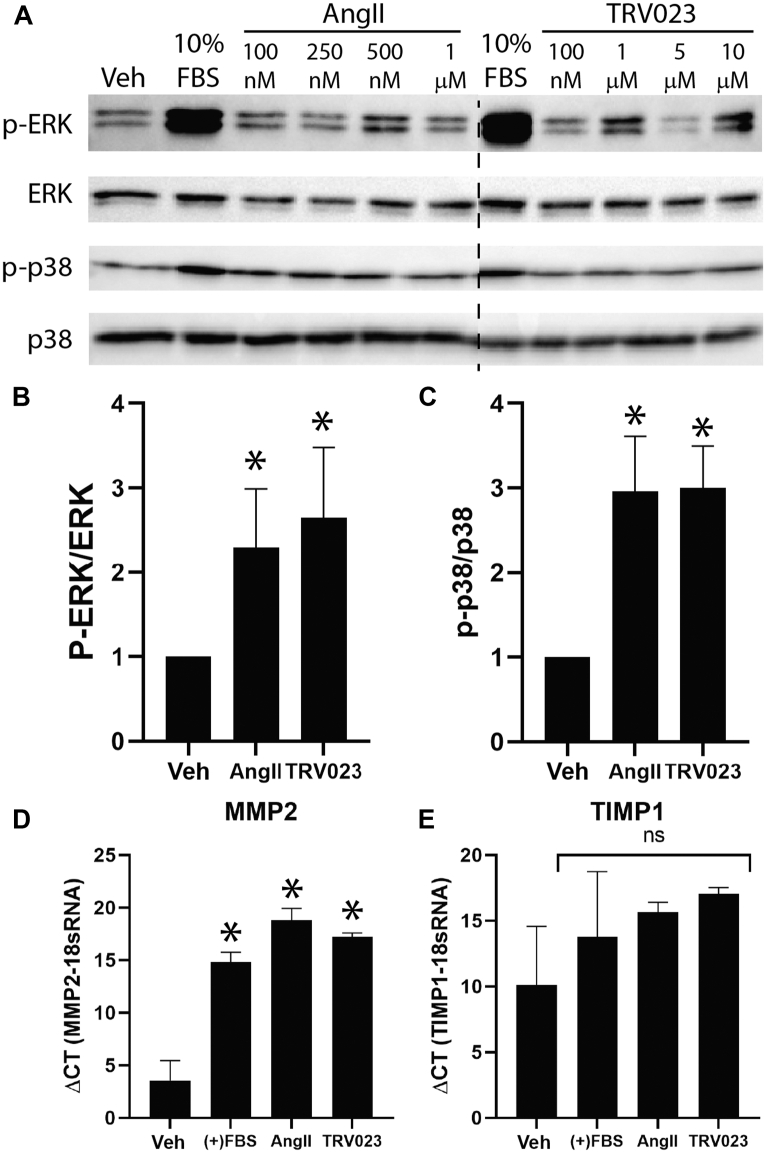
AngII and TRV023 Activate MAP Kinases in PASMCs Isolated From Patients With PH PASMCs isolated from patients with PH were cultured and stimulated with AngII or TRV023 for analyzing phosphorylation of ERK and p38 at 10 and 20 minutes, respectively. **(A)** Representative immunoblot demonstrating AngII- and TRV023-induced phosphorylation of ERK and p38 activation in PASMCs isolated from patients with PH. Quantitation of **(B)** phospho-ERK and **(C)** phospho-p38 MAP kinases from n = 3 biological repeats of patients with PH ([AngII] = 500 nM, [TRV023] = 5 μM). Real-time PCR of targets important in regulation of cell migration demonstrate that AngII and TRV023 both increase levels of **(D)** MMP2 with no significant expression of **(E)** TIMP1 expression. ∗*P* < 0.05 by 1-way analysis of variance from vehicle-treated samples. ERK = extracellular signal-regulated kinase; FBS = fetal bovine serum; MAP = mitogen-activated protein; MMP = matrix metalloproteinase; PASMC = pulmonary artery smooth muscle cell; PCR = polymerase chain reaction; pERK = phospho-ERK; p-p38 = phospho-p38; TIMP = tissue inhibitor of metallopeptidases; Veh = vehicle; other abbreviations as in Figure 1.

Based on our transcriptomic findings, we hypothesized that the MAP kinase signaling cascade is activated in MCT PH rats treated with TRV023 or AngII. Previous studies have shown AngII-mediated transient activation of ERK at 2 to 5 minutes and remains elevated for 60 minutes in vascular smooth muscle cells (36, 37, 38). To confirm if TRV023 also mediates activation of MAPK signaling in PASMCs from patients with PH, we analyzed phosphorylation of ERK and p38 MAP kinases. PASMCs were grown at 60% to 70% confluency and starved for 24 hours and treated with increasing concentration of AngII or TRV023 for 10 minutes for ERK phosphorylation and 20 minutes for p-38 phosphorylation. Immunoblots showed increased phosphorylation of ERK (Figures 4A and 4B) and p38 (Figures 4A and 4C) with increasing concentration of AngII as well as TRV023. The expression and activation of MMPs can also be of significant importance in vascular remodeling (8,39). To determine whether AT_1_R activation directly involves in the activation of MMPs, consistent with our previous results, we found that AngII and TRV023 increased the messenger RNA expression of MMP-2 (Figure 4D) but not TIMP-1 in PH PASMCs (Figure 4E), consistent with an effect that would promote vascular remodeling. We did not observe any other significant differences between groups in expression of similar markers (Supplemental Figure 5). These data suggest β-arrestin–biased AT_1_R stimulation contributes to pulmonary vascular remodeling through multiple signaling axes.

### β-arrestin–mediated AT_1_R signaling stimulates human PASMC proliferation and migration

**Figure 5:**
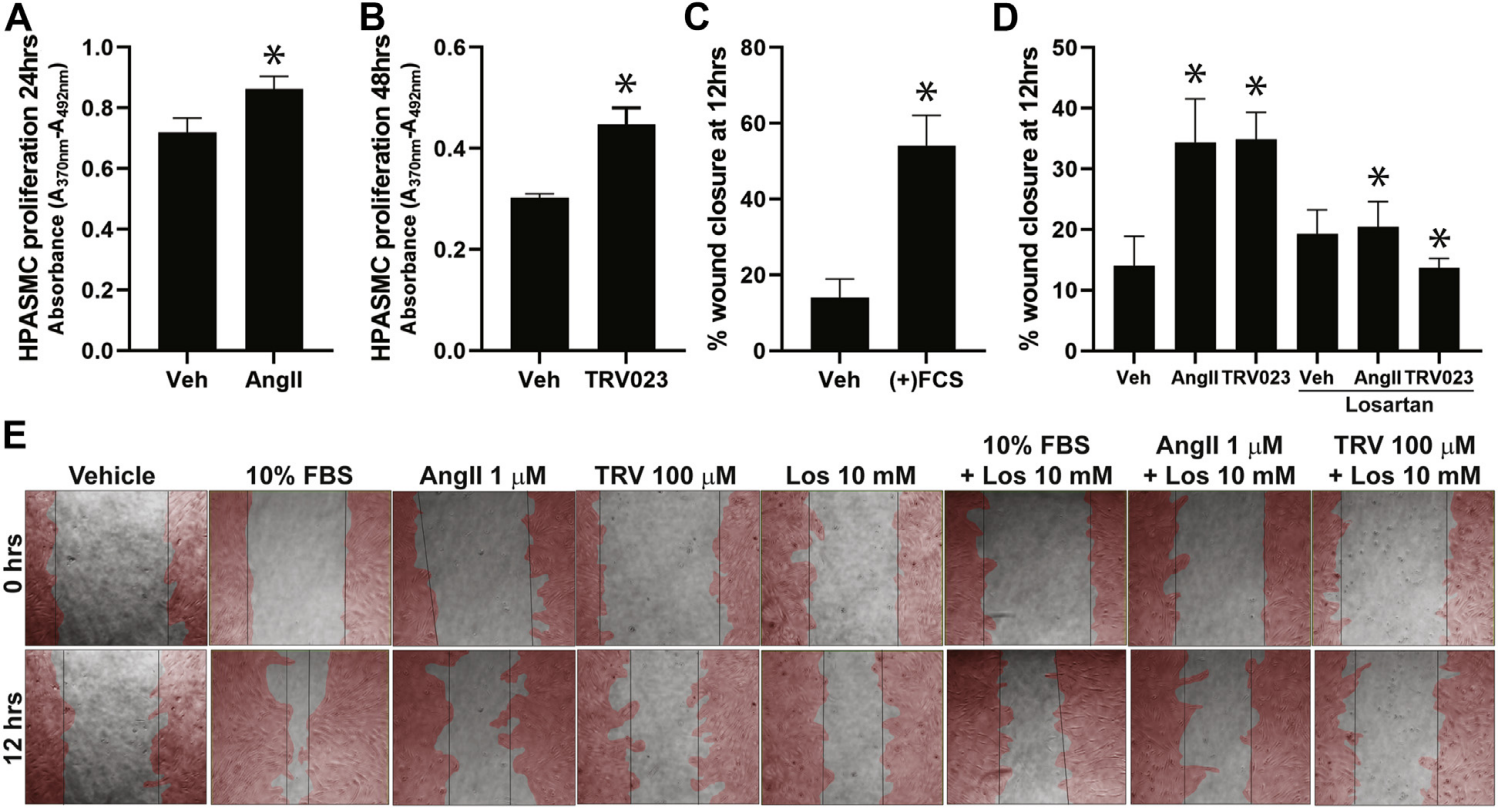
AngII and TRV023 Promote PH-PASMC Proliferation and Migration **(A)** AngII-mediated proliferation of PASMCs at 24 hours. ∗*P* < 0.05 versus vehicle. **(B)** TRV023-mediated proliferation of PASMCs at 48 hours. ∗*P* < 0.05 versus vehicle. Quantification of **(C)** FBS-induced (∗*P* < 0.05 versus vehicle) and **(D)** AngII- and TRV023-induced PASMC migration at 12 hours (∗*P* < 0.05 versus vehicle). AngII- and TRV023-mediated migration was blocked by losartan (∗*P* < 0.05 versus AngII and TRV023 with no losartan). **(D)** Representative live cell imaging of AngII and TRV023 induced PASMC migration at 0 and 12 hours. Manual cell outlines are shown in **red**. Statistical analysis was performed by 1-way analysis of variance. n = 3 biological repeats of PASMCs from patients with PH. Abbreviations as in Figures 1 and 4.

AngII stimulates vascular smooth muscle cell proliferation and migration by activating tyrosine kinases and MAPK signaling, and increasing intracellular Ca^2+^ levels that are regulated by both G proteins and β-arrestins downstream of the AT_1_R (40, 41, 42). To examine the effects of TRV023 on PASMC proliferation and migration in vitro, we performed BrdU proliferation and migration (wound healing scratch assays) in PASMCs isolated from PH patients. PH PASMCs were stimulated with AngII and TRV023 and proliferation monitored after stimulation for AngII (Figure 5A) and TRV023 (Figure 5B) (41). To examine the effects of AngII and TRV023 on PASMC migration, PASMCs were subjected to in vitro scratch wound healing assay (Figure 5C) in the presence or absence of AngII, TRV023, or losartan. The scratch assay showed that both AngII and TRV023 increased PASMC migration, whereas losartan significantly decreased the rate of migration induced by both ligands (Figures 5D and 5E). These findings demonstrate that AngII and TRV023 both promote PASMC proliferation and migration via the AT_1_R, consistent with a phenotype of pulmonary vascular remodeling in vivo.

## Discussion

Despite current treatments, PAH is a devastating disease with a prognosis worse than many cancers and a 3-year survival of 68% to 70% (43,44). Therefore, new strategies are urgently needed to directly address the pathological pulmonary vascular remodeling that underlies PAH. Current PAH therapies such as prostacyclin receptor agonists and endothelin receptor antagonists act as pulmonary vasodilators through their effects on vascular smooth muscle cells (45,46). These agents have been proven to be effective in PAH, with benefits in exercise capacity and clinical endpoints (47, 48, 49); however, it is largely unknown to what extent the benefits of these drugs are related to their vasodilatory or antiproliferative properties. Here, we demonstrated that an AT_1_R β-arrestin–biased agonist that acts as a vasodilator (by blocking G protein–mediated signaling) while promoting signaling through β-arrestins did not have a beneficial effect and led to adverse pulmonary vascular remodeling and worse outcomes compared with losartan, an AT_1_R antagonist that blocked both G protein– and β-arrestin–mediated signaling. Unfortunately, we could not compare the responses of these drugs to strongly G protein–biased AT_1_R agonists, which have not been developed at this time (21). Similarly, the lack of biased agonists targeting the endothelin and prostacyclin receptors currently precludes a similar study of those receptors. As there are currently no described biased agonists of GPCR ligands at the endothelin or prostacyclin receptors that could be tested in our studies, we are limited in our conclusions regarding current PAH therapies. However, our findings suggest that the beneficial aspects of current PAH-specific therapies are not only through the acute effects on vasoconstriction but through their long-term effects on pulmonary vascular remodeling. Indeed, it is likely that the beneficial effects of current PAH therapies act both as vasodilators and anti-proliferative agents.

In contrast to our study in PAH, previous work has shown that β-arrestin–biased AT_1_R agonists have a number of distinct physiological effects compared with AngII in other models of cardiovascular disease. For example, acute infusion of AngII markedly increased mean arterial pressure in male spontaneously hypertensive rats, accompanied by a reduction of glomerular filtration rate (48), whereas TRV023 blocked the acute infusion of AngII-induced hypertension state in a dose-dependent manner in mice (16). Likewise, chronic administration of AngII in rats has been shown to induce LV hypertrophy, which can be blunted by coadministration of TRV023 or losartan (24). Consistent with these results, we found that acute infusion of AngII, but not of TRV023, increased LV and RV systolic pressures. The β-arrestin–biased agonist TRV120027 (15) competitively antagonized AngII-induced mean arterial pressure elevation while stimulating cardiac contractility as determined by load-independent measurements (15,24), whereas the AT_1_R antagonist telmisartan decreased mean arterial pressure and cardiac contractility (15). TRV120027 has also been shown cause a neonatal-specific sustained positive inotropic effect without increasing heart rate, an effect thought to be mediated by its activation of L-type calcium channels (50). TRV023 has been shown to increase LV contractility and promote cell survival in mice, the effects of which are dependent on β-arrestin (16). Taken together, conventional and β-arrestin–biased AT_1_R agonists are pharmacologically distinct in vivo.

In preclinical models of left heart failure, TRV120027 decreased mean arterial pressure, right atrial pressure, pulmonary capillary wedge pressure, and systemic and renal vascular resistances, while increasing cardiac output and renal blood flow (18,51). In a mouse model of familial dilated cardiomyopathy, TRV023 increased cardiac performance, suggesting that AT_1_R-biased ligands may prove to be a novel inotropic approach in familial dilated cardiomyopathy (17). Furthermore, in line with the preclinical findings, infusion of TRV120027 was safe and well tolerable and leads to the reductions of mean arterial pressure in healthy volunteers with sodium intake restriction (52). In contrast to left heart failure, PAH is characterized by a marked increase in RV afterload and subsequent dilation, fibrosis, and RV failure. The mechanisms underlying the development of RV failure secondary to PAH remain an area of active investigation. RV function determines the symptoms of patients with PAH and is one of the most important factors for survival. Unlike LV dysfunction, there are currently no therapies that directly target the RV in PAH (53,54). Here, we found that losartan significantly improved survival compared with vehicle, AngII, or TRV023. In contrast to its effects in models of LV dysfunction, TRV023 failed to prolong survival compared with control or AngII, and TRV023 did not improve the hemodynamics or enhance cardiac performances in RV PV loop analyses. These results suggest that β-arrestin–biased AT_1_R ligands are not beneficial for targeting the RV or pulmonary vasculature in PAH. Our observations are largely consistent with a previous study that showed no differences in RV hypertrophy between AT_1_R antagonist losartan and vehicle-treated rats with MCT-induced PAH (4); however, in contrast to our findings, they observed RV afterload was reduced in losartan-treated rats with PAH, without affecting RV contractility (4). This inconsistency may be due to differences in treatment and follow-up time between these studies.

The lack of cardiac effects of TRV023 led us to examine potential AT_1_R-mediated pulmonary vascular remodeling. Pulmonary vascular remodeling in PAH is characterized by endothelial dysfunction (27), clonal expansion of apoptosis-resistant endothelial cells, and smooth muscle cell proliferation, along with a complex interplay between adventitial fibroblasts, perivascular inflammatory cells, and the extracellular matrix (1,55). In MCT-induced PAH rats, there were no discernible changes in PA wall media thickness across different treatments. Intriguingly, although both AngII and TRV023 increased Ki-67 levels in pulmonary arterioles, TRV023 increased it to a greater extent, consistent with an increase in SMC cell ploidy, as can be seen in hypertrophy, or with SMC proliferation. This disparity could be explained by increased apoptosis as quantified by the elevation of cleaved caspase-3 in AngII-treated pulmonary arteries, consistent with a previous study that showed that AngII markedly increased proapoptotic activity in the suprarenal aortas in *ApoE* knockout mice (56). Our transcriptomic data demonstrated that the TRV023 group had higher levels of transcripts associated with cell proliferation. These results are consistent with TRV023 promoting pulmonary vascular remodeling in MCT-induced PAH rats.

AT_1_R overexpression in patients with idiopathic PAH correlates with the activation of MAPK and SRC signaling compared with controls (4) and AngII treatment selectively induces the proliferation of SMCs isolated from patients with idiopathic PAH (4). Consistent with this, AngII and TRV023 activated ERK1/2 and SRC signaling in U2OS and HEK cells overexpressing rat AT_1_R (15). Here we found that a number of signaling effectors regulated by MAPK signaling pathways were increased in MCT rats treated with AngII or TRV023 compared with vehicle or losartan in the lung transcriptome analysis. We further verified our results in vitro that AngII and TRV023 stimulated the proliferation and migration of PASMCs and induced the phosphorylation of ERK1/2 and p38 in PH PASMCs, effects that were blunted by losartan. As SMC proliferation and migration can be induced by alterations in the composition of the extracellular matrix from MMP activation (39), we then tested the effects of AngII and TRV023 on transcript levels of MMP-2 and TIMP-1 in PH PASMCs. We found that both AngII and TRV023 increased MMP-2 levels, likely through the activation of Erk1/2 signaling by AngII and TRV023, as it has been demonstrated that tryptase activates the Erk1/2 signaling cascade to induce the production of fibronectin and MMP-2 in PASMCs (57). Taken together, β-arrestin–biased ligands likely promote PASMC proliferation and migration through multiple pathways, contributing to pulmonary vascular remodeling in PH.

### Study Limitations

As noted in the Discussion, we could not study an AT1R G protein-biased agonist to test its effects alone on the PAH phenotype, as no strongly G protein-biased AT1R agonists have been identified. Similarly, we could not test the effects of biased agonists targeting current PAH drug targets as biased endothelin and prostacyclin agonists have not been reported. The MCT PH model is associated with high mortality levels, which limited our ability to assess hemodynamics in the setting of severe PH.

## Conclusions

In conclusion, our findings demonstrate that activating β-arrestin–biased AT_1_R signaling promotes vascular remodeling and is undesirable in PAH. This suggests that the development of new PAH therapies at targets that have previously been considered to only promote vasodilation, such as GPCRs, can still reverse pulmonary vascular remodeling in PAH through mitigating apoptosis resistance and angioproliferative lung pathology.

Perspectives **COMPETENCY IN MEDICAL KNOWLEDGE:** PAH is a disease of pulmonary vascular remodeling with endothelial cell apoptosis, smooth muscle proliferation, and fibroblast activation that results in pulmonary vascular obstruction and right heart failure. The beneficial effect of current therapies for PAH is thought to be based primarily on their role as vasodilators, although any long-term benefit of these therapies is likely related to their promotion of pulmonary vascular reverse remodeling. At this time, there are few data to support a role for these therapies in promoting long-term pulmonary vascular reverse remodeling. To address this knowledge gap, we took advantage of “biased agonists” of the AT_1_R, a receptor that is known to play a role in pulmonary vascular remodeling. Although the AT_1_R is not targeted clinically in PAH, other GPCRs, such as the type A endothelin and prostacyclin receptor, are drug targets in PAH. We found that the AT_1_R “biased agonist” TRV023, which acts as a pulmonary vasodilator but also promotes cellular proliferation, promoted similar levels of abnormal pulmonary vascular remodeling as the vasoconstrictor AngII, effects that could be blocked by the antagonist losartan. Thus, this study suggests that the beneficial effect of current PAH therapies is through their long-term effects on pulmonary vascular reverse remodeling, although they may also have a short-term benefit from promoting pulmonary vasodilation as well.

**TRANSLATIONAL OUTLOOK:** Currently there are no described biased agonists targeting the endothelin or prostacyclin receptors, which are well-validated targets in the treatment of PAH. Biased agonists of these and other receptors may have distinct effects from balanced GPCR agonists and antagonists in PAH. For example, prostacyclin receptor agonists are frequently limited in their dosage due to side effects associated with acute vasodilation. The development of a biased agonist that could promote signaling through pathways important in reverse remodeling while preventing vasodilation could be an approach to limit side effects of these medications while reaching higher therapeutic doses of drug.

## Funding Support and Author Disclosures

The Mandel Foundation funded the Duke Cardiovascular Research Center Physiology Core. Dr Rajagopal was funded by a Gilead Research Scholars in Pulmonary Hypertension, Burroughs Wellcome Career Award for Medical Scientists Award, and K08HL114643. The authors have reported that they have no relationships relevant to the contents of this paper to disclose.

## References

[1] Morrell N.W., Adnot S., Archer S.L. (2009). Cellular and molecular basis of pulmonary arterial hypertension. J Am Coll Cardiol.

[2] Humbert M., Guignabert C., Bonnet S. (2019). Pathology and pathobiology of pulmonary hypertension: state of the art and research perspectives. Eur Respir J.

[3] Rubin L.J. (2002). Therapy of pulmonary hypertension: the evolution from vasodilators to antiproliferative agents. Am J Respir Crit Care Med.

[4] de Man F.S., Tu L., Handoko M.L. (2012). Dysregulated renin-angiotensin-aldosterone system contributes to pulmonary arterial hypertension. Am J Respir Crit Care Med.

[5] Morrell N.W., Atochina E.N., Morris K.G., Danilov S.M., Stenmark K.R. (1995). Angiotensin converting enzyme expression is increased in small pulmonary arteries of rats with hypoxia-induced pulmonary hypertension. J Clin Invest.

[6] Morrell N.W., Danilov S.M., Satyan K.B., Morris K.G., Stenmark K.R. (1997). Right ventricular angiotensin converting enzyme activity and expression is increased during hypoxic pulmonary hypertension. Cardiovasc Res.

[7] Morrell N.W., Morris K.G., Stenmark K.R. (1995). Role of angiotensin-converting enzyme and angiotensin II in development of hypoxic pulmonary hypertension. Am J Physiol.

[8] Okada M., Harada T., Kikuzuki R., Yamawaki H., Hara Y. (2009). Effects of telmisartan on right ventricular remodeling induced by monocrotaline in rats. J Pharmacol Sci.

[9] Rockman H.A., Koch W.J., Lefkowitz R.J. (2002). Seven-transmembrane-spanning receptors and heart function. Nature.

[10] Murasawa S., Mori Y., Nozawa Y. (1998). Angiotensin II type 1 receptor-induced extracellular signal-regulated protein kinase activation is mediated by Ca2+/calmodulin-dependent transactivation of epidermal growth factor receptor. Circ Res.

[11] Rakesh K., Yoo B., Kim I.M., Salazar N., Kim K.S., Rockman H.A. (2010). beta-Arrestin-biased agonism of the angiotensin receptor induced by mechanical stress. Sci Signal.

[12] Mehta P.K., Griendling K.K. (2007). Angiotensin II cell signaling: physiological and pathological effects in the cardiovascular system. Am J Physiol Cell Physiol.

[13] Forrester S.J., Booz G.W., Sigmund C.D. (2018). Angiotensin II signal transduction: an update on mechanisms of physiology and pathophysiology. Physiol Rev.

[14] Pfeffer M.A., Swedberg K., Granger C.B. (2003). Effects of candesartan on mortality and morbidity in patients with chronic heart failure: the CHARM-Overall programme. Lancet.

[15] Violin J.D., DeWire S.M., Yamashita D. (2010). Selectively engaging beta-arrestins at the angiotensin II type 1 receptor reduces blood pressure and increases cardiac performance. J Pharmacol Exp Ther.

[16] Kim K.S., Abraham D., Williams B., Violin J.D., Mao L., Rockman H.A. (2012). beta-Arrestin-biased AT1R stimulation promotes cell survival during acute cardiac injury. Am J Physiol Heart Circ Physiol.

[17] Tarigopula M., Davis R.T., Mungai P.T. (2015). Cardiac myosin light chain phosphorylation and inotropic effects of a biased ligand, TRV120023, in a dilated cardiomyopathy model. Cardiovasc Res.

[18] Boerrigter G., Lark M.W., Whalen E.J., Soergel D.G., Violin J.D., Burnett J.C. (2011). Cardiorenal actions of TRV120027, a novel ss-arrestin-biased ligand at the angiotensin II type I receptor, in healthy and heart failure canines: a novel therapeutic strategy for acute heart failure. Circ Heart Fail.

[19] Wingler L.M., Elgeti M., Hilger D. (2019). Angiotensin analogs with divergent bias stabilize distinct receptor conformations. Cell.

[20] Rajagopal K., Whalen E.J., Violin J.D. (2006). Beta-arrestin2-mediated inotropic effects of the angiotensin II type 1A receptor in isolated cardiac myocytes. Proc Natl Acad Sci U S A.

[21] Strachan R.T., Sun J.P., Rominger D.H. (2014). Divergent transducer-specific molecular efficacies generate biased agonism at a G protein-coupled receptor (GPCR). J Biol Chem.

[22] Rajagopal S., Ahn S., Rominger D.H. (2011). Quantifying ligand bias at seven-transmembrane receptors. Mol Pharmacol.

[23] Pogoriler J.E., Rich S., Archer S.L., Husain A.N. (2012). Persistence of complex vascular lesions despite prolonged prostacyclin therapy of pulmonary arterial hypertension. Histopathology.

[24] Monasky M.M., Taglieri D.M., Henze M. (2013). The beta-arrestin-biased ligand TRV120023 inhibits angiotensin II-induced cardiac hypertrophy while preserving enhanced myofilament response to calcium. Am J Physiol Heart Circ Physiol.

[25] Li Q., Dale W.E., Hasser E.M., Blaine E.H. (1996). Acute and chronic angiotensin hypertension: neural and nonneural components, time course, and dose dependency. Am J Physiol.

[26] Ma Z., Mao L., Rajagopal S. (2016). Hemodynamic characterization of rodent models of pulmonary arterial hypertension. J Vis Exp.

[27] Antonia A.L., Gibbs K.D., Trahair E.D. (2019). Pathogen evasion of chemokine response through suppression of CXCL10. Front Cell Infect Microbiol.

[28] Sutendra G., Dromparis P., Paulin R. (2013). A metabolic remodeling in right ventricular hypertrophy is associated with decreased angiogenesis and a transition from a compensated to a decompensated state in pulmonary hypertension. J Mol Med.

[29] Gomez-Arroyo J.G., Farkas L., Alhussaini A.A. (2012). The monocrotaline model of pulmonary hypertension in perspective. Am J Physiol Lung Cell Mol Physiol.

[30] Zhang J., Liu Y., Zheng Y. (2020). TREM-2-p38 MAPK signaling regulates neuroinflammation during chronic cerebral hypoperfusion combined with diabetes mellitus. J Neuroinflammation.

[31] Liu D.D., Lu J.M., Zhao Q.R., Hu C., Mei Y.A. (2016). Growth differentiation factor-15 promotes glutamate release in medial prefrontal cortex of mice through upregulation of T-type calcium channels. Sci Rep.

[32] Li S., Ma Y.M., Zheng P.S., Zhang P. (2018). GDF15 promotes the proliferation of cervical cancer cells by phosphorylating AKT1 and Erk1/2 through the receptor ErbB2. J Exp Clin Cancer Res.

[33] Wilson J.L., Yu J., Taylor L., Polgar P. (2015). Hyperplastic growth of pulmonary artery smooth muscle cells from subjects with pulmonary arterial hypertension is activated through JNK and p38 MAPK. PLoS One.

[34] Yamboliev I.A., Gerthoffer W.T. (2001). Modulatory role of ERK MAPK-caldesmon pathway in PDGF-stimulated migration of cultured pulmonary artery SMCs. Am J Physiol Cell Physiol.

[35] Jiang B., Yamamura S., Nelson P.R., Mureebe L., Kent K.C. (1996). Differential effects of platelet-derived growth factor isotypes on human smooth muscle cell proliferation and migration are mediated by distinct signaling pathways. Surgery.

[36] Duff J.L., Berk B.C., Corson M.A. (1992). Angiotensin II stimulates the pp44 and pp42 mitogen-activated protein kinases in cultured rat aortic smooth muscle cells. Biochem Biophys Res Commun.

[37] Tsuda T., Kawahara Y., Ishida Y., Koide M., Shii K., Yokoyama M. (1992). Angiotensin II stimulates two myelin basic protein/microtubule-associated protein 2 kinases in cultured vascular smooth muscle cells. Circ Res.

[38] Eguchi S., Matsumoto T., Motley E.D., Utsunomiya H., Inagami T. (1996). Identification of an essential signaling cascade for mitogen-activated protein kinase activation by angiotensin II in cultured rat vascular smooth muscle cells. Possible requirement of Gq-mediated p21ras activation coupled to a Ca2+/calmodulin-sensitive tyrosine kinase. J Biol Chem.

[39] Bendeck M.P., Zempo N., Clowes A.W., Galardy R.E., Reidy M.A. (1994). Smooth muscle cell migration and matrix metalloproteinase expression after arterial injury in the rat. Circ Res.

[40] Maron B.A., Leopold J.A. (2014). The role of the renin-angiotensin-aldosterone system in the pathobiology of pulmonary arterial hypertension (2013 Grover Conference series). Pulm Circ.

[41] Morrell N.W., Upton P.D., Higham M.A., Yacoub M.H., Polak J.M., Wharton J. (1998). Angiotensin II stimulates proliferation of human pulmonary artery smooth muscle cells via the AT1 receptor. Chest.

[42] Zhang F., Hu Y., Xu Q., Ye S. (2010). Different effects of angiotensin II and angiotensin-(1-7) on vascular smooth muscle cell proliferation and migration. PLoS One.

[43] Hurdman J., Condliffe R., Elliot C.A. (2012). ASPIRE registry: Assessing the Spectrum of Pulmonary hypertension Identified at a REferral centre. Eur Respir J.

[44] Farber H.W., Miller D.P., Poms A.D. (2015). Five-year outcomes of patients enrolled in the REVEAL Registry. Chest.

[45] Lai Y.C., Potoka K.C., Champion H.C., Mora A.L., Gladwin M.T. (2014). Pulmonary arterial hypertension: the clinical syndrome. Circ Res.

[46] Sitbon O., Morrell N. (2012). Pathways in pulmonary arterial hypertension: the future is here. Eur Resp Rev.

[47] Zamanian R.T., Kudelko K.T., Sung Y.K., Perez V.J., Liu J., Spiekerkoetter E. (2014). Current clinical management of pulmonary arterial hypertension. Circ Res.

[48] Elmarakby A.A., Bhatia K., Crislip R., Sullivan J.C. (2016). Hemodynamic responses to acute angiotensin II infusion are exacerbated in male versus female spontaneously hypertensive rats. Physiol Rep.

[49] Barst R.J., Gibbs J.S., Ghofrani H.A. (2009). Updated evidence-based treatment algorithm in pulmonary arterial hypertension. J Am Coll Cardiol.

[50] Kashihara T., Kawagishi H., Nakada T. (2020). beta-arrestin-biased AT1 agonist TRV027 causes a neonatal-specific sustained positive inotropic effect without increasing heart rate. J Am Coll Cardiol Basic Trans Science.

[51] Boerrigter G., Soergel D.G., Violin J.D., Lark M.W., Burnett J.C. (2012). TRV120027, a novel beta-arrestin biased ligand at the angiotensin ii type i receptor, unloads the heart and maintains renal function when added to furosemide in experimental heart failure. Circ Heart Fail.

[52] Soergel D.G., Subach R.A., Cowan C.L., Violin J.D., Lark M.W. (2013). First clinical experience with TRV027: pharmacokinetics and pharmacodynamics in healthy volunteers. J Clin Pharmacol.

[53] Ryan J.J., Archer S.L. (2014). The right ventricle in pulmonary arterial hypertension: disorders of metabolism, angiogenesis and adrenergic signaling in right ventricular failure. Circ Res.

[54] Franco V. (2012). Right ventricular remodeling in pulmonary hypertension. Heart Fail Clin.

[55] Rabinovitch M. (2012). Molecular pathogenesis of pulmonary arterial hypertension. J Clin Invest.

[56] Wang Y.X., Martin-McNulty B., da Cunha V. (2005). Fasudil, a Rho-kinase inhibitor, attenuates angiotensin II-induced abdominal aortic aneurysm in apolipoprotein E-deficient mice by inhibiting apoptosis and proteolysis. Circulation.

[57] Kwapiszewska G., Markart P., Dahal B.K. (2012). PAR-2 inhibition reverses experimental pulmonary hypertension. Circ Res.

