# Supplementary material for: β-Arrestin–Mediated Angiotensin II Type 1 Receptor Activation Promotes Pulmonary Vascular Remodeling in Pulmonary Hypertension
Source: JACC Basic Transl Sci. 2021 Nov 22;6(11):854–69. doi: 10.1016/j.jacbts.2021.09.006 (PMC8617598; doi:10.1016/j.jacbts.2021.09.006)
Supplement: Supplemental Figures 1–5 and Supplemental Tables 1 and 2 [file mmc1.pdf]

Supplementary Appendix for

β-arrestin-mediated Angiotensin II type 1 Receptor Activation Promotes Pulmonary Vascular Remodeling in Pulmonary Hypertension

Running Title: β-arrestin-mediated AT_1R signaling promotes PAH

Zhiyuan Ma[1†], Gayathri Viswanathan[1†], Mason Sellig[2], Chanpreet Jassal[4], Issac Choi[1], Aditi Garikipati[1], Xinyu Xiong[1], Nour Nazo[1], Sudarshan Rajagopal[1, 3, *]

Supplemental Figure 1. Hemodynamic effects of chronic infusion of AT1R ligands on right ventricular pressure-volume loop parameters in MCT PH rats. 2

Supplemental Figure 2. Effects of chronic infusion of AT1R ligands on LV hypertrophy in MCT rats. 3

Supplemental Figure 3. DAPI, Ki67, and α-smooth muscle actin (SMA) staining of lung samples from vehicle, AngII, TRV023 and losartan-treated MCT PH rats. 4-5

Supplemental Figure 4. Confirmation of RNA sequencing by qPCR. 6

Supplemental Figure 5. AngII and TRV023 alter the composition of the extracellular matrix in PASMCs isolated from PH patients. 7

Supplemental Table 1. List of PCR primers - human PH PASMCs. 8

Supplemental Table 2. List of PCR primers - rat MCT PH. 9

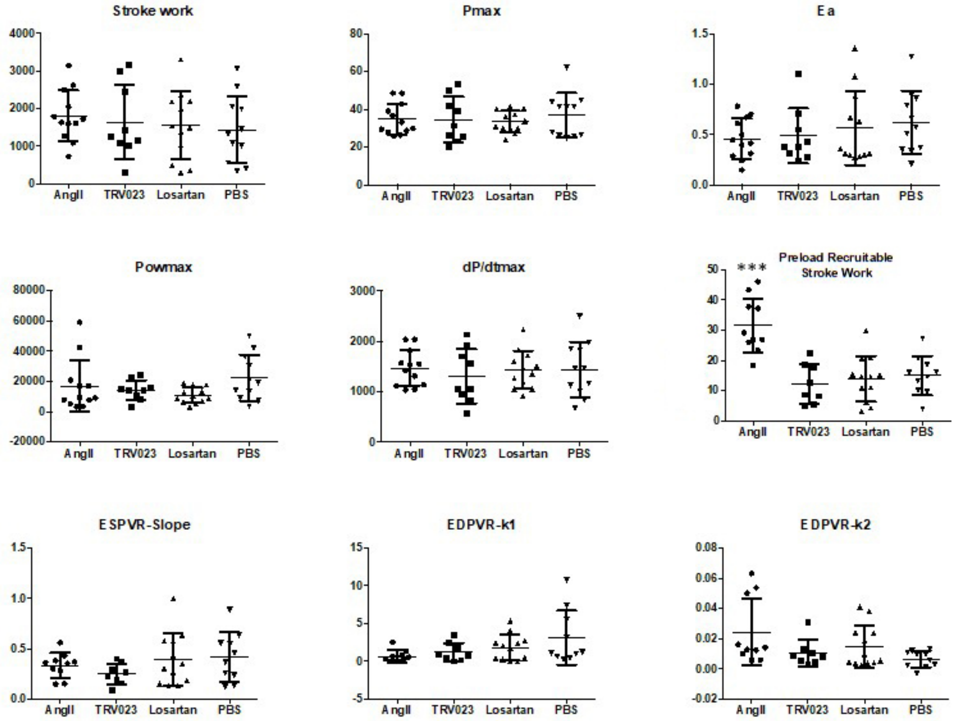

Supplemental Figure 1. Hemodynamic effects of chronic infusion of AT_1R ligands on right ventricular pressure-volume loop parameters in MCT PH rats. Chronic infusion of AngII, TRV023 or losartan in MCT rats resulted in no significant changes in the majority of hemodynamic parameters from pressure-volume loop analysis of the right ventricle. The only significant difference that was noted was an increase in preload recruitable stroke work in AngII-treated rats (***, p < 0. 001). Maximal pressure (Pmax), End-systolic elastance (Ea), Maximal power (Pmax), Maximum of Change in Pressure / Change in time (dP/dtmax), end-systolic pressure volume relationship slope (ESPVR-slope), end-diastolic pressure volume relationship coefficient 1 (EDPVR-k1), end-diastolic pressure volume relationship coefficient 2 (EDPVR-k2). Statistical analysis was performed by one-way ANOVA and Tukey’s multiple comparisons test.

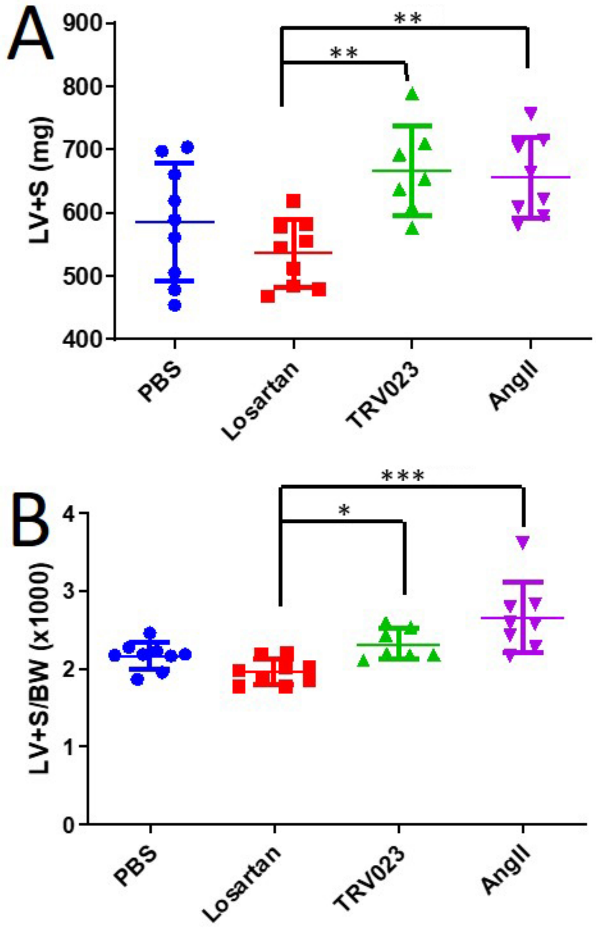

Supplemental Figure 2. Effects of chronic infusion of AT1R ligands on LV hypertrophy in MCT rats. Treatment with TRV023 and AngII both induced LV hypertrophy compared to losartan. (**A**) as assessed by left ventricular + septum (LV+S) weight and (**B**) LV+S corrected by rat body weight. Statistical analysis was performed by one-way ANOVA and Tukey's multiple comparisons test. (*, $p < 0.05$, **, $p < 0.01$, ***, $p < 0.001$)

Supplemental Figure 3. DAPI, Ki67, and α-smooth muscle actin (SMA) staining of lung samples from replicates of vehicle, AngII, TRV023 and losartan-treated MCT PH rats.

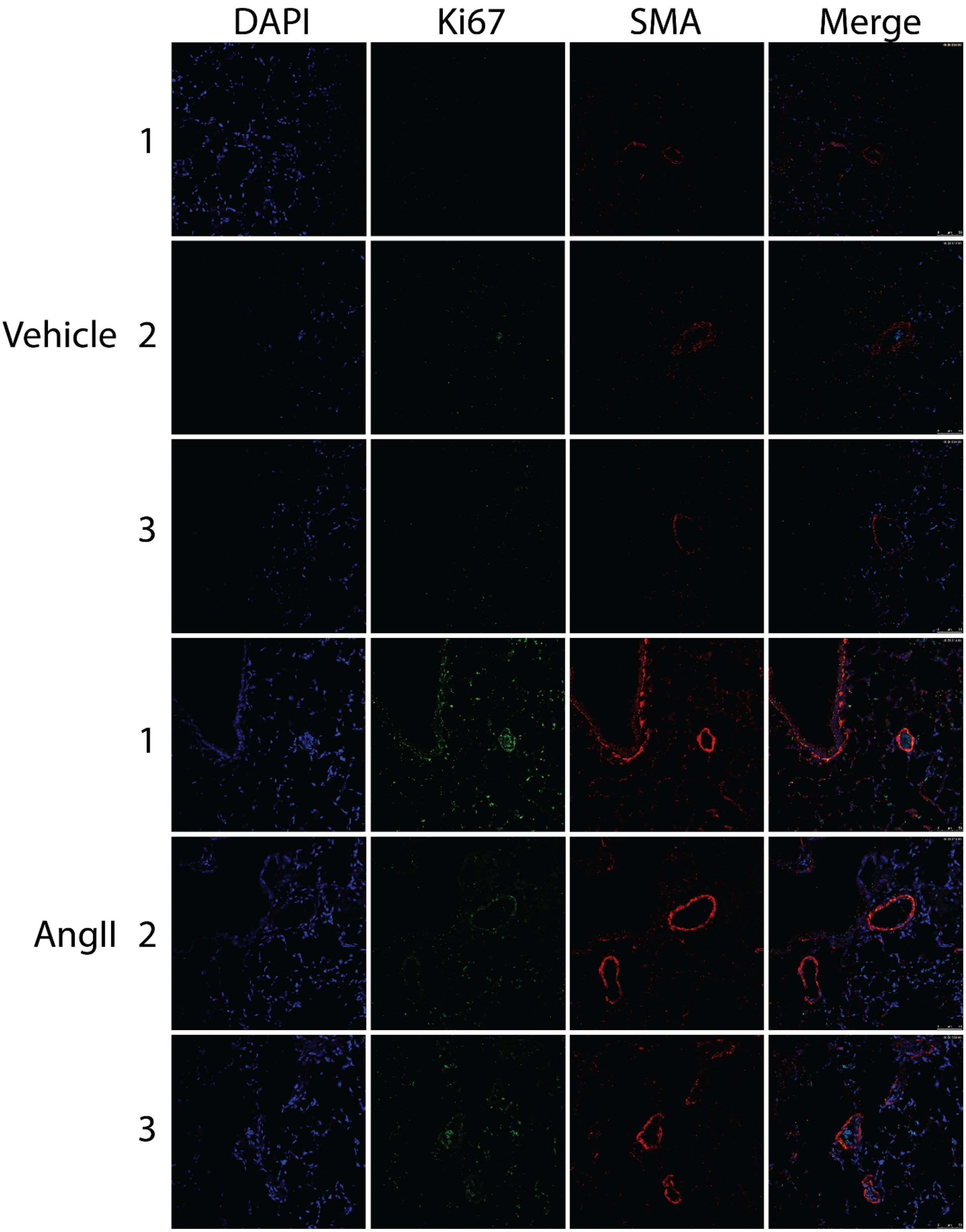

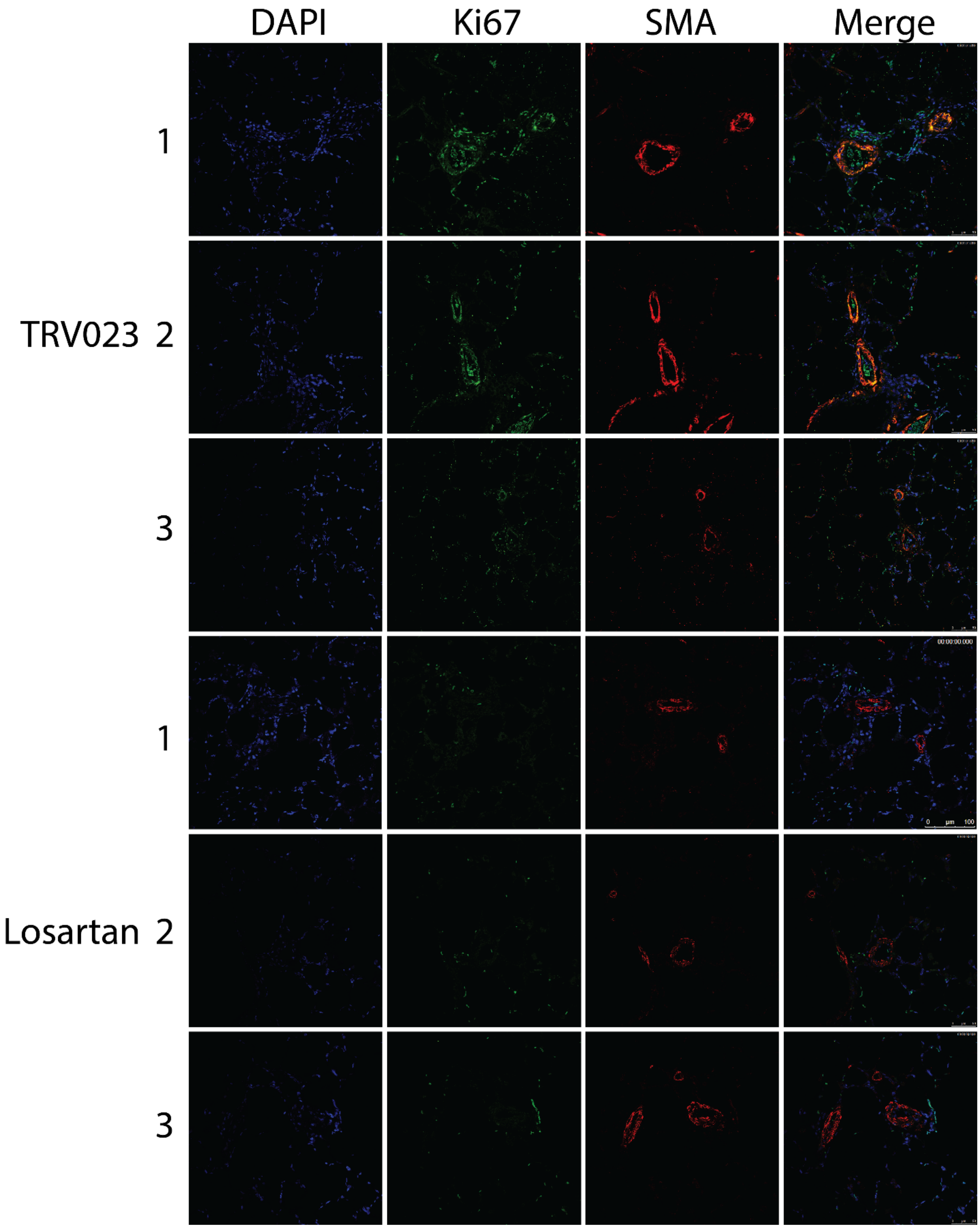
DAPI
Ki67
SMA
Merge
1
TRV023 2
3
1
Losartan 2
3
0 µm 100

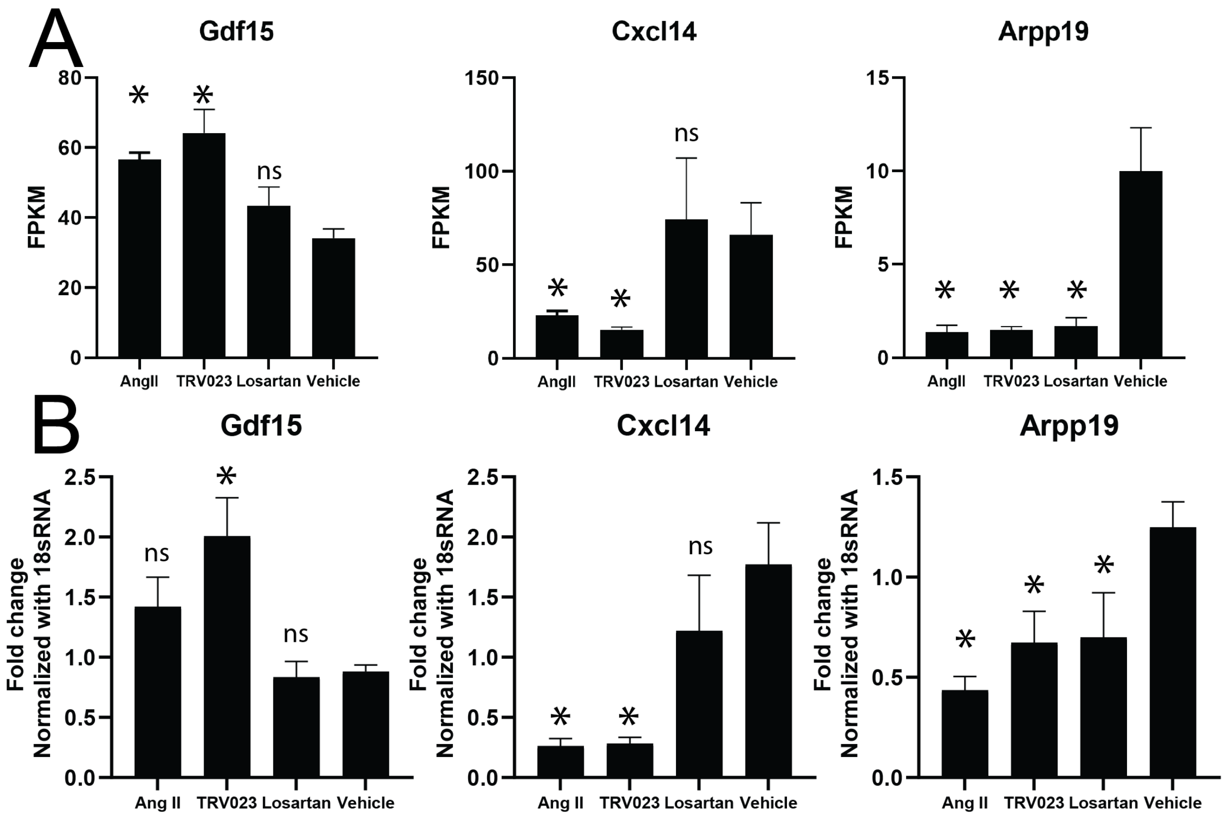

Supplemental Figure 4. Confirmation of RNA sequencing results by qPCR. A, RNA sequencing and **B**, qPCR of specific transcripts that were differentially expressed between AngII, TRV023, Losartan and vehicle-treated samples from MCT PH rats. *, p < 0.05 versus vehicle treated MCT rats. FPKM – fragments per kilobase of exon model per million reads mapped.

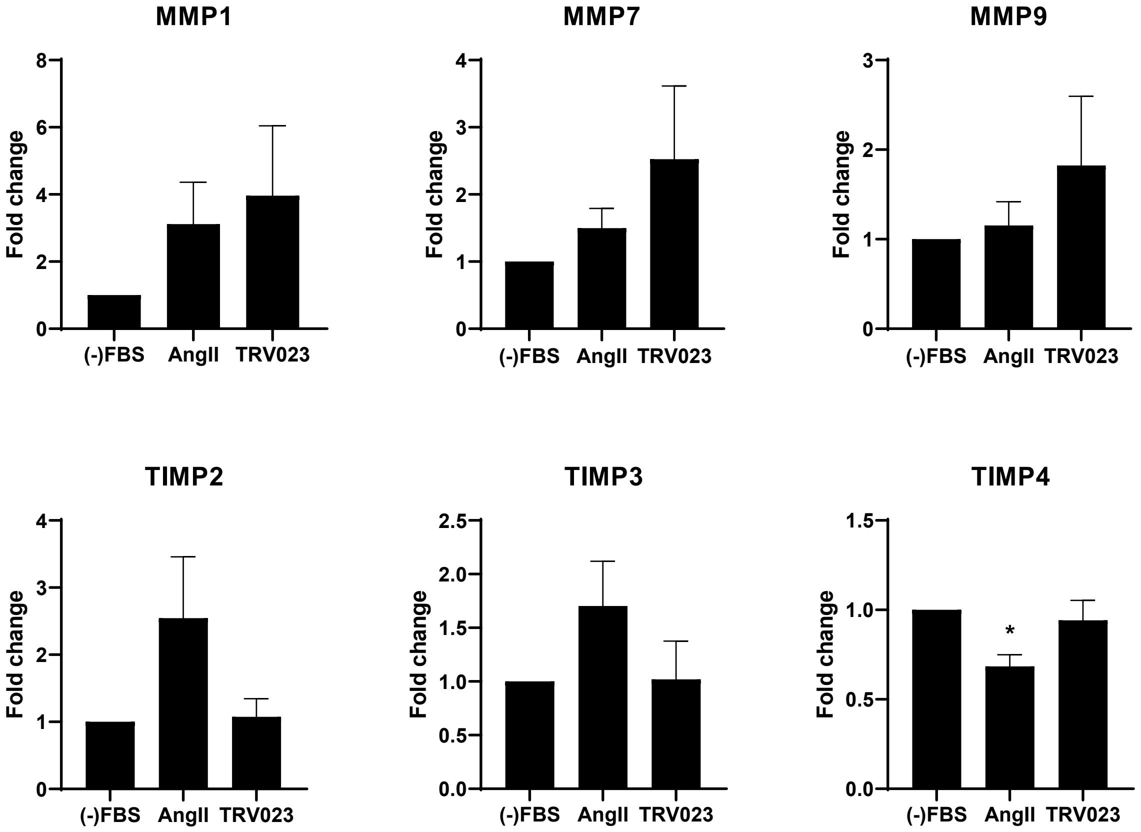

Supplemental Figure 5. AngII and TRV023 alter the composition of the extracellular matrix in PASMCs isolated from PH patients. PASMCs isolated from n=3 biological repeats of PH patients were cultured and stimulated with 500nM AngII or 5 μM TRV023 for analyzing mRNA expression of MMPs and TIMPs. *, $p < 0.05$ by one-way ANOVA from vehicle treated samples.

Supplemental Table 1. List of PCR primers - human PH PASMCs.

**Gene**	**Forward primer**	**Reverse primer**
Mmp9	GCACGA CGT CTT CCA GTA CC	CAG GAT GTC ATA GGT CAC GTA GC
Mmp7	GTATGGGACATTCCTCTGATCC	CCAATGAATGAATGAATG GATG
Mmp1	CCTAGCTACACCTTCAGTGG	GCCCAGTACTTATTCCCTTT
Mmp2	ATGACAGCTGCACCACTGAG	CTCCTGAATGCCCTTGATGT
Mmp9	TACCCTATGTACCGCTTCAC	GAACAAATACAGCTGGTTCC
18S rRNA	GTAACCCGTTGAACCCCATT	CCATCCAATCGGTAGTAGCG
Timp1	TGACATCCGGTTCGTCTACA	GTTTGCAGGGGATGGATAAA
Timp2	CCGCAACAGGCGTTTTGCAA	TCACTTCTCTTGATGCAGGC
Timp3	TTCTGCAACTCCGACATCGT	ATGCAGGCGTAGTGTTTGGA
Timp4	CACTACCATCTGAACTGTGGCTG	GCTTTCGTTCCAACAGCCAGTC

Supplemental Table 2. List of PCR primers - rat MCT PH.

Gene	Forward primer	Reverse primer
Gdf15	CCTCTCTGAGTCCCAACTCAAC	AATCGCACCTCTGGACTGAGTA
Cxcl14	AAGCTGGAAATGAAGCCAAAG	GTTCCAGGCGTTGTACCATTT
Arpp19	AGATTGCAGAAAGGGCAAAAG	TAGCAACAAGGGATGGTTTCC
18S rRNA	ACTCAACACGGGAAACCTCA	AATCGCTCCACCAACTAAGA
